# Digital gene expression profiling analysis of duodenum transcriptomes in SD rats administered ferrous sulfate or ferrous glycine chelate by gavage

**DOI:** 10.1038/srep37923

**Published:** 2016-11-30

**Authors:** Zhao Zhuo, Shenglin Fang, Qiaoling Hu, Danping Huang, Jie Feng

**Affiliations:** 1 Key Laboratory of Molecular Animal Nutrition, Ministry of Education, College of Animal Science, Zhejiang University, Hangzhou, 310058, P. R. China

## Abstract

The absorption of different iron sources is a trending research topic. Many studies have revealed that organic iron exhibits better bioavailability than inorganic iron, but the concrete underlying mechanism is still unclear. In the present study, we examined the differences in bioavailability of ferrous sulfate and ferrous glycinate in the intestines of SD rats using Illumina sequencing technology. Digital gene expression analysis resulted in the generation of almost 128 million clean reads, with expression data for 17,089 unigenes. A total of 123 differentially expressed genes with a |log2(fold change)| >1 and q-value < 0.05 were identified between the FeSO_4_ and Fe-Gly groups. Gene Ontology functional analysis revealed that these genes were involved in oxidoreductase activity, iron ion binding, and heme binding. Kyoto Encyclopedia of Genes and Genomes pathway analysis also showed relevant important pathways. In addition, the expression patterns of 9 randomly selected genes were further validated by qRT-PCR, which confirmed the digital gene expression results. Our study showed that the two iron sources might share the same absorption mechanism, and that differences in bioavailability between FeSO_4_ and Fe-Gly were not only in the absorption process but also during the transport and utilization process.

Iron is an essential trace element for life that is involved in various biologic processes, including oxygen transport, energy metabolism, DNA biosynthesis and oxidative phosphorylation^1^,^2^. It lacks a controlled excretion mechanism; therefore, iron homeostasis in the body is primarily regulated by iron absorption from the duodenal epithelium and its recycling in macrophages and other tissue stores^3^,^4^. Iron is potentially toxic, and its accumulation in the body results in the generation of reactive oxygen species (ROS)^5^,^6^. However, iron deficiency is a prevalent nutritional problem affecting humans and animals^7^. Dietary iron supplementation has long been used to prevent and treat iron deficiency in animals^8^,^9^, but different iron sources vary in their bioavailability.

It has been reported that iron chelated with amino acid or protein has good bioavailability in animals^10^,^11^,^12^. Recent studies have shown that ferrous glycinate (Fe-Gly) is more effective in animal production than ferrous sulfate (FeSO_4_)^13^,^14^. Fe-Gly is absorbed more efficiently and utilized faster than FeSO_4_, and in addition, the expression of intestinal transport proteins differs in the presence of these two iron sources^15^. However, the concrete mechanism underlying the absorption of these two iron sources is still unknown.

Next generation sequencing (NGS) techniques are effective methods that have dramatically improved the speed and efficiency of the identification of novel genes^16^,^17^. Digital gene expression (DGE), a tag-based transcriptome sequencing method, is one such technique that can be applied to analyze quantitative gene expression and to compare expression profiles without being affected by potential bias, thereby enabling sensitive and accurate transcriptome profiling^18^,^19^.

In this study, we applied RNA sequencing technology to assess the absorption mechanisms of different iron sources in the intestines of Sprague-Dawley (SD) rats. Because iron is mainly absorbed in the duodenum^20^,^21^, only duodenal samples were examined in DGE analysis. By assembling and annotating the transcriptome sequences identified in these samples, and analyzing their gene expression profiles, we were able to identify differentially expressed genes in response to the two iron sources. The results of our DGE analysis have provided preliminary information regarding the differences between FeSO_4_ and Fe-Gly absorption in SD rats.

## Results

### Iron status of SD rats

**Figure 1:**
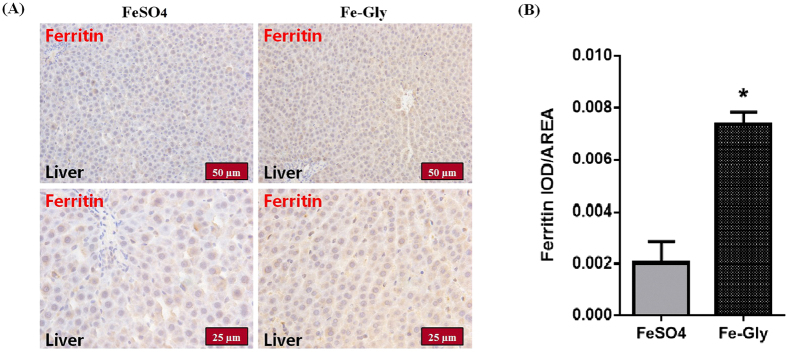
Immunohistochemical staining of ferritin in the liver. (**A**) Liver biopsies in different magnifications (50 μm and 25 μm) are shown for each group. (**B**) Image-Pro Plus 6.0 was used to determine integrated optical density (IOD) values, from which the mean density was calculated (IOD/AREA). *Represents a significant difference in the mean value between the two groups at P < 0.05.

After two weeks of treatment of the SD rats by intragastric administration of the different iron sources, the animals’ body weights did not differ between the FeSO_4_ and Fe-Gly groups (Table 1). In addition, no differences in the hematological parameters were observed between the two groups (Table 2). The serum total iron binding capacity (TIBC) were similar between the groups, but the serum iron (SI) levels were significantly different (P-value < 0.05, Table 3). The Fe-Gly group exhibited a higher serum iron concentration than the FeSO_4_ group; therefore, transferrin saturation (TAST) was also increased (P-value < 0.05). The immunohistochemical staining of ferritin in the liver also differed between the two groups (Fig. 1). The liver biopsies of the Fe-Gly group in different magnifications (50 μm and 25 μm) showed increased positive staining, indicating enhanced ferritin deposition in the liver. These results were confirmed by calculation of the mean density (P-value < 0.05).

### Analysis of DGE libraries

To detect differences in absorption between FeSO_4_ and Fe-Gly, RNA-seq of duodenal samples was performed using the Illumina sequencing platform. Three individual samples were included for each group, and they were marked as C1, C2, and C3 and T1, T2, and T3, respectively. The main characteristics of the six libraries are summarized in Table 4. The C1, C2, C3, T1, T2, and T3 libraries contained 20,446,968, 20,983,958, 23,325,694, 21,951,812, 21,586,433, and 22,458,258 raw reads, respectively. After removing adaptor, ambiguous and low-quality sequences, 19,720,103, 20,414,770, 22,897,985, 21,550,142, 21,172,114, and 22,056,148 clean reads were remained. The percentage of clean reads among raw reads was greater than 96%.

### Mapping reads to the transcriptome

For gene expression profiling, the sequencing reads from the six libraries were aligned to a reference database, which consisted of the *Rattus norvegicus* genome, using TopHat v2.0.9. More than 95% of the clean reads mapped to this database (Table 5). In particular, 17,935,148 (90.95%), 18,282,777 (89.56%), 20,841,830 (91.02%), 19,627,268 (91.08%), 19,087,487 (90.15%), and 19,997,125 (90.66%) reads from the C1, C2, C3, T1, T2, and T3 libraries, respectively, uniquely mapped to the reference database.

### Analysis of differential gene expression

**Figure 2:**
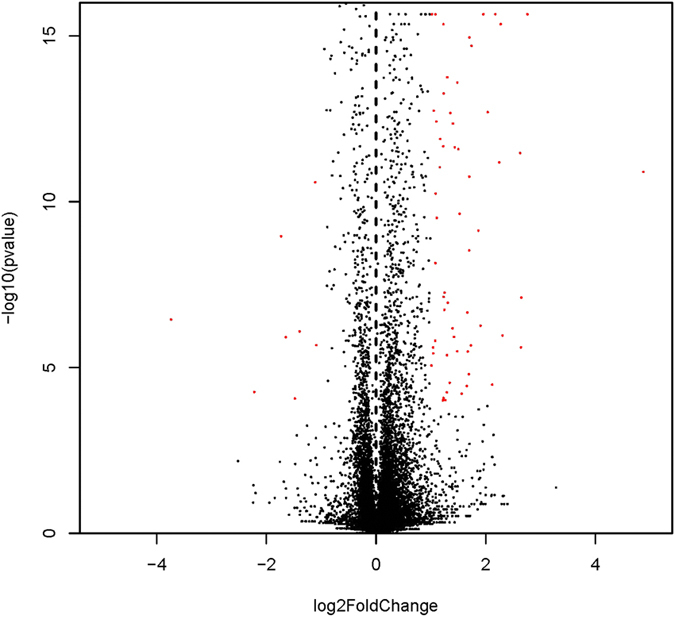
Volcano plot of differentially expressed genes. The abscissa represents the fold changes in gene expression, which were calculated as Fe-Gly (mean)/FeSO_4_ (mean); the “mean” is the mean of three biological replicates. The ordinate represents the statistical significance of the variations in gene expression. The red dots represent significantly differentially expressed genes.

**Figure 3:**
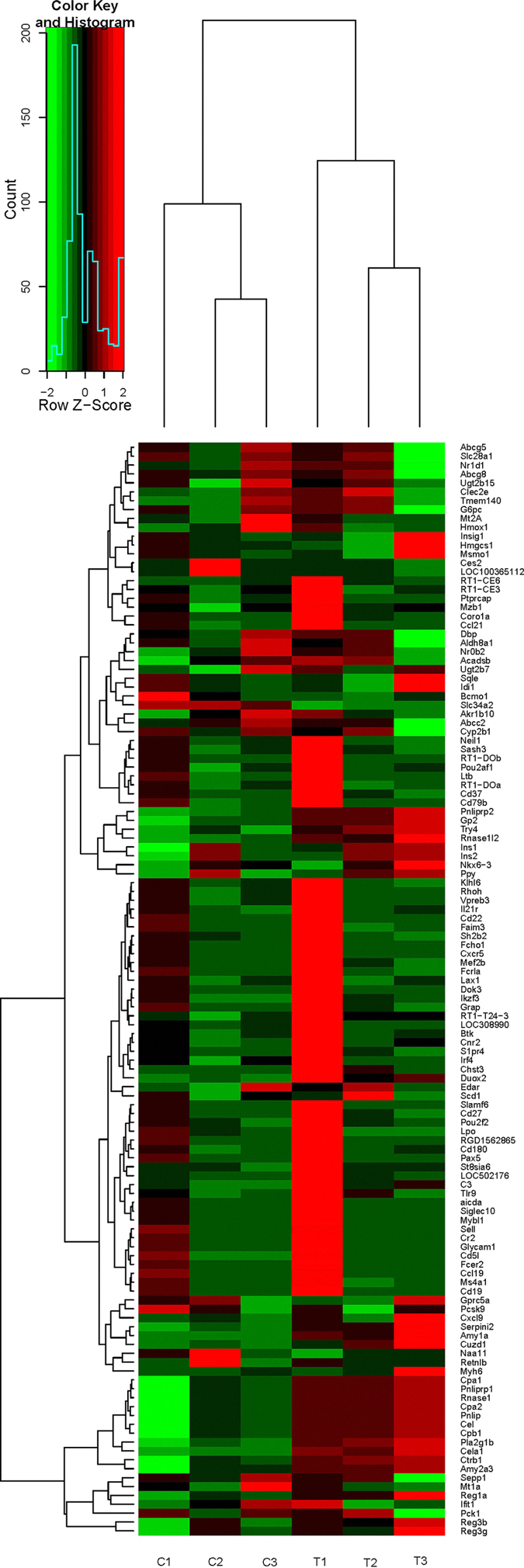
Heatmap of differentially expressed genes. C1, C2, and C3: control group, namely the FeSO_4_ group; T1, T2, and T3: treatment group, namely the Fe-Gly group.

For analysis of gene expression, the number of unambiguous clean tags for each gene was calculated and normalized to the RPKM value. To increase the accuracy of the measured expression levels for further analyses, data from three biological replicates were merged, and RPKM values were calculated based on the merged dataset (ref. Table S1). To identify differentially expressed genes, a |log2(fold change)| >1 and q-value < 0.05 were used as standards. A volcano plot was generated to visualize the distribution of expressed genes between the groups (Fig. 2), and the red dots in this plot represent differentially expressed genes. The distribution of differentially expressed genes is depicted in the heatmap shown in Fig. 3. There were 123 differentially expressed genes in total, including 83 up-regulated and 40 down-regulated genes (ref. Table S2).

### Functional analysis of differentially expressed genes

**Figure 4:**
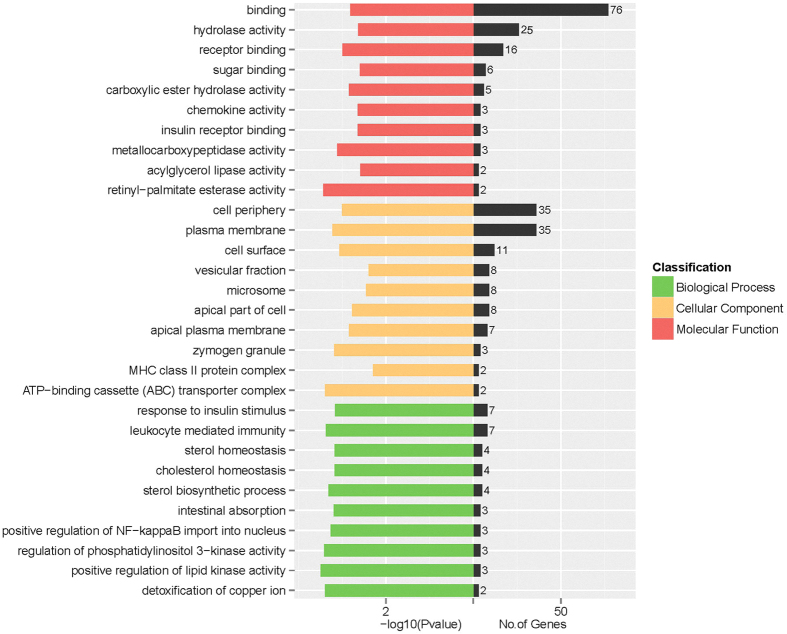
GO functional enrichment analysis. The differentially expressed genes between the FeSO_4_ and Fe-Gly groups were classified based on Gene Ontology. Only a portion of the results are shown; for the complete dataset, please see ref. Table S3.

The differentially expressed genes were considered to be associated with changes in physiological function in the body. According to the Gene Ontology (GO) classification system, the 123 differentially expressed genes were classified into three main functional categories: biological process, cellular component and molecular function (ref. Table S3). Genes involved in the response to stimulus, metabolic process, response to chemical stimulus, and response to organic substance were predominant in the biological process category. In addition, plasma membrane, endomembrane system, membrane fraction and microsome were the predominant enriched terms in the cell components category. Moreover, a significant proportion of the genes were involved in binding, catalytic activity, receptor binding, and oxidoreductase activity in the category of molecular function. Iron ion binding, monooxygenase activity and heme binding were also notable enriched terms, as they are closely related to iron metabolism. A portion of the GO analysis results is shown in Fig. 4.

**Figure 5:**
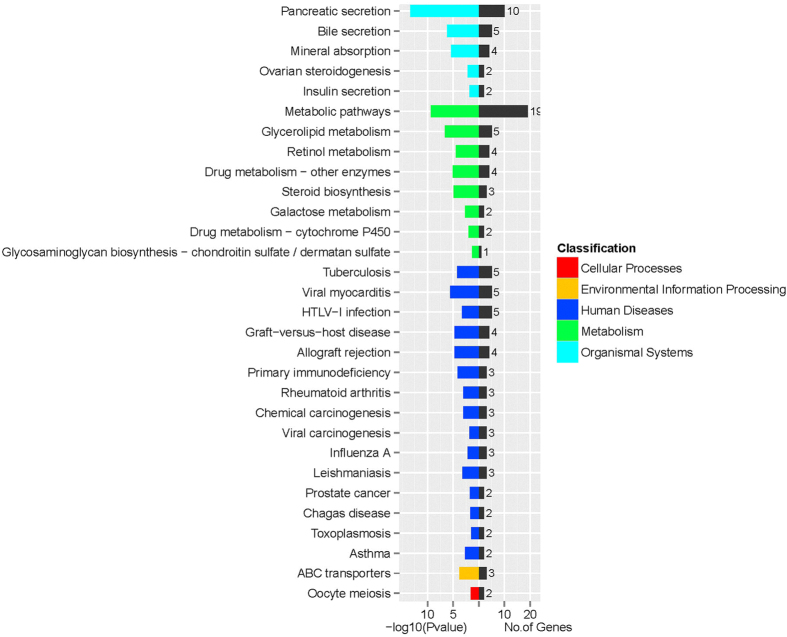
KEGG pathway analysis. The enriched pathways among the differentially expressed genes were identified by KEGG analysis. Only a portion of the results are shown; for the complete dataset, please see ref. Table S4.

Kyoto Encyclopedia of Genes and Genomes (KEGG) pathway analysis of the 123 differentially expressed genes was also performed (ref. Table S4). The results indicated that these genes were mainly classified into six biochemical pathways: metabolism (24, 19.5%), genetic information processing (20, 16.3%), environmental information processing (3, 2.4%), cellular processes (2, 1.6%), organismal systems (56, 45.5%) and human diseases (23, 18.7%). The associated secondary pathways included pancreatic secretion, mineral absorption, insulin secretion, metabolic pathways, steroid biosynthesis, and ABC transporters (Fig. 5).

### Confirmation of differentially expressed genes by qRT-PCR

**Figure 6:**
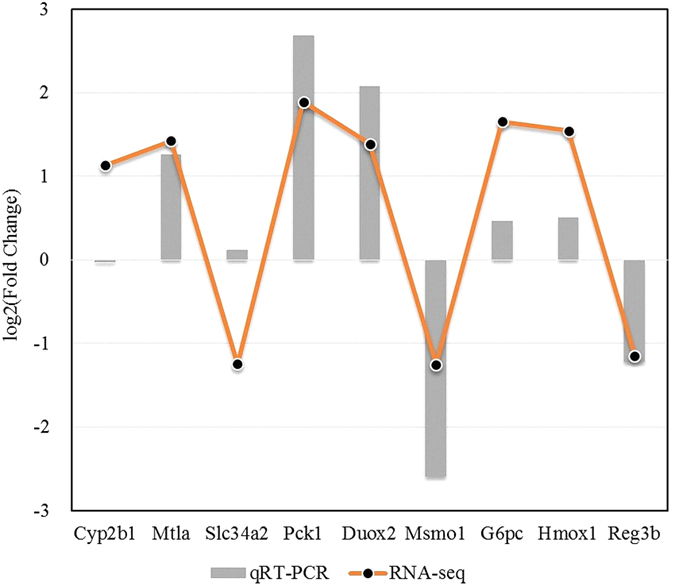
Gene expression determined by RNA-seq and qRT-PCR. qRT-PCR validation of nine differentially expressed genes between the FeSO_4_ and Fe-Gly groups. The data were normalized to the expression of GAPDH, and the fold changes were calculated as Fe-Gly/FeSO_4_.

To validate the tag-mapped genes, the transcript levels of 9 differentially expressed genes identified by RNA-seq were examined by real-time quantitative PCR (Fig. 6). The PCR primers used are shown in Supplementary Table S5. The qRT-PCR results revealed significant differences in the expression of six genes (Mt1a, Pck1, Duox2, Msmo1, Hmox1, and Reg3b) in line with the digital gene expression data (P-value < 0.05). In addition, one gene (G6pc) was found to be non-significantly up-regulated (P-value > 0.05) by qRT-PCR, which was also in accord with the RNA-seq results. Only two genes (Cyp2b1, and Slc34a2) did not show consistent expression between the qRT-PCR and RNA-seq data. However, our experimental results are still valid because RNA-seq has a known false positive rate^22^.

## Discussion

Although many studies have shown that ferrous glycinate exhibits better bioavailability than ferrous sulfate^15^,^23^,^24^, the mechanism of its high bioavailability is still unclear. In the present study, we found that gavage of SD rats with FeSO_4_ or Fe-Gly did not affect their growth performance over a relatively short time period (two weeks), but that the SI level was significantly increased in the Fe-Gly-treated rats group. SI is the amount of circulating iron that is bound to transferrin^23^, and it reflects iron absorption in the intestinal tract. These results are consistent with those of our previous study showing that Fe-Gly is absorbed better than FeSO_4_^15^. TIBC reflects the blood’s capacity to bind iron with transferrin^25^, and it often increases under iron-deficient conditions. In our study, the TIBC was similar between the two groups, but TAST was significantly increased in the Fe-Gly group. These results suggested that more iron was transported into the bodies of the Fe-Gly-treated rats. However, administration of the two iron sources did not significantly influence the Hb level. This result is reasonable because the Hb level is usually within the normal range in humans and animals, and remains constant because it is regulated by the iron homeostasis system^26^. The liver is a reliable response criterion for the mineral status^27^, and ferritin is a protein that functions in iron storage *in vivo*^28^. The results showed increased positive staining for ferritin in the liver biopsies of the Fe-Gly group, suggesting that Fe-Gly was more easily absorbed and transported into the rats’ bodies than FeSO_4_.

To clarify the molecular mechanisms of FeSO_4_ and Fe-Gly absorption, the transcriptomes of duodenal samples obtained from SD rats administered one of the two iron sources by gavage were sequenced using the Illumina platform. This approach provides a new method to study the absorption of different iron sources using the recently developed RNA-seq technology. In total, almost 128 million clean reads were obtained. Approximately 17,089 unigenes were assembled, of which 100% were annotated (ref. Table S1). A total of 123 differentially expressed genes with a |log2(fold change)| >1 and q-value < 0.05 were identified between the FeSO_4_ and Fe-Gly groups. To validate the differentially expressed genes identified by RNA-seq, the expression levels of 9 genes were confirmed by qRT-PCR. Comparison of the results obtained using the two methods revealed similar trends of up-regulation and down-regulation.

According to the GO classifications, the differentially expressed genes were involved in oxidoreductase activity, iron ion binding, monooxygenase activity, and heme binding activity. Cyp2b1, Hmox1, Duox2, and Msmo1 were the main genes associated with these GO molecular function terms. In addition, KEGG pathway analysis of the 123 differentially expressed genes revealed that, metabolic pathways, pancreatic secretion, and cytokine-cytokine receptor interaction were the most highly enriched terms. We also focused on the differentially expressed genes related to the mineral absorption pathway, HIF-1 signaling pathway and ABC transporters.

The mineral absorption pathway was associated with a bunch of these elements absorption (e.g., for Ca, P, K, Na, Fe, Cu, Zn, Mn). They are one of the five fundamental groups of nutrients that clearly required for life, but most are quite toxic when present at higher than normal concentrations. Thus, there is a physiologic challenge of supporting efficient but limited absorption. In many cases intestinal absorption is a key regulatory step in mineral homeostasis. In the present study, three genes involved in the mineral absorption pathway were markedly up-regulated by Fe-Gly gavage, that are Hmox1, Mt1a and Mt2A. Heme oxygenase 1 (Hmox1) is involved in the release of iron from heme^29^. There are studies shown that rats cannot absorb heme iron as efficiently as humans do, and they don’t require intestinal Hmox1 for dietary heme iron assimilation^30^,^31^. But glycine was one of the important substrate in the process of heme synthesis^32^, the increased Hmox1 expression of Fe-Gly group in our experiment indicated that Fe-Gly was more closely linked to intracellular heme metabolism than FeSO_4_. MTs are small (6–10 kDa), cysteine-rich (33%) metalloproteins that catalyze redox reactions and contain metal binding sites. Although they are mainly involved in the homeostasis of physiological Zn^2+^, they still exhibit the capacity to bind iron because they are thiolate-rich biomolecules^33^. Considering that the expression of Mt1a and Mt2A was significantly increased in the Fe-Gly group, we speculate that a much larger amount of iron was indeed absorbed into the intestinal epithelia of the rats in this group. Duodenal *Npt 2b* (Slc34a2) primarily mediates intestinal Pi absorption^34^, which was also the differentially expressed gene involved in the mineral absorption pathway. In our experiment, the down-regulated Slc34a2 by Fe-Gly gavage seem to indicate that the intracellular iron content will affect intestine Pi absorption.

As a master regulator of the hypoxia-signaling pathway, the HIF-1 signaling pathway has been conserved throughout evolution in species ranging from *Caenorhabditis elegans* to *Homo sapiens*, these pathways activate the expression of similar (or homogenous) genes, resulting in similar physical and biochemical responses, including oxygen sensing, oxygen transport, angiogenesis, erythropoiesis, and heme metabolism^35^. In this study, insulin 1 (Ins1) and insulin 2 (Ins2), which are involved in the HIF-1 signaling pathway, were down-regulated in the Fe-Gly group. Because the insulin level is increased under iron-deficient conditions^36^, it seems that the SD rats in the Fe-Gly group maintained better iron status than those in the FeSO_4_ group.

ATP-binding cassette (ABC) transporters belong to one of the largest known protein families, and they are widespread in bacteria, archaea, and eukaryotes^37^. They couple ATP hydrolysis to the active transport of a wide variety of substrates, such as ions, sugars, lipids, sterols, peptides, proteins, and drugs^38^. Heme and iron siderophores have been shown to be transported across the cytoplasmic membrane by ABC transporters^39^. In this study, three genes involved in the ABC transporter pathway were up-regulated in the Fe-Gly group, suggesting that Fe-Gly more effectively increased the activity of ABC transporters. Thus, the two iron sources may have had different fates after being absorbed by the intestinal epithelium.

To our knowledge, the intestinal absorption of inorganic iron often begins with the conversion of Fe^3+^ to Fe^2+^ by duodenal cytochrome b (DcytB), which is a membrane-associated ferrireductase^40^. Then, the reduced Fe^2+^ is transported across the apical membrane by divalent metal transporter 1 (DMT1/SLC11A2)^41^,^42^. The absorbed iron is either stored intracellularly for subsequent use or transported into the circulation by the only known iron export protein, ferroportin (FPN1/SLC40A1)^43^. We had previously thought that the difference in biological efficiency between FeSO_4_ and Fe-Gly might be attributed to their differing mechanisms of absorption. However, there were few differentially expressed genes related to iron metabolism between the two groups (Table 6).

Our previous cell experiments demonstrated that, in the same concentration, FeSO_4_ had more free iron ion than Fe-Gly^44^. Fe^2+^ is easily oxidized to be Fe^3+^, and Fe^3+^ is apparently less effective in the body^45^,^46^. In addition, the environment of intestine is complex, many factors can affect iron absorption. Ferrous glycinate is a relatively stable compound, chelated with glycine ligand can protect iron from inhibitors in the intestine and keep it soluble and readily available^47^,^48^. Our results are more inclined to support that the two iron sources are absorbed through the inorganic iron way, their bioavailability differences might mainly due to differ in the absorption rate of iron in the intestine.

## Conclusion

In the present study, we examined the absorption differences between FeSO_4_ and Fe-Gly in SD rats. Digital gene expression profiling analysis based on Illumina sequencing technology provided comprehensive information on iron metabolism. There were 123 significantly differentially expressed genes in total, including 83 up-regulated and 40 down-regulated genes. GO functional analysis revealed that these genes were related to oxidoreductase activity, iron ion binding, and heme binding. KEGG pathway analysis showed that they were also involved in important pathway, such as mineral absorption, the HIF-1 signaling pathway and ABC transporters. In addition, the expression patterns of 9 genes were further validated by qRT-PCR, confirming the digital gene expression results. Our study indicated that the two iron sources might share the same absorption mechanism, and that FeSO_4_ and Fe-Gly might differ not only in their absorption process but also in their transport and utilization process.

## Methods

### Animals and experimental design

All of the animal experiments were approved by the Animal Ethics Committee of Zhejiang University. The experimental procedures were performed in strict accordance with the Guidelines for the Care and Use of Laboratory Animals in China. This study was conducted at the Laboratory Animal Center of Zhejiang University. After two days of pre-feeding, twenty-four SD rats (males; 4 weeks old) were randomly assigned to receive one of the two treatments. Every day, the rats in each treatment group were perfused with 1 mL FeSO_4_ or Fe-Gly (80 mg/L as iron). The experiment lasted for two weeks.

The SD rats were reared in a clean standard room. Their diet was formulated according to the International Standards of Experimental Animals AIG-93G (purchased from Slack Experimental Animals LLC, Shanghai; for composition of the basal diet, see ref. Table S6). The temperature and relative humidity in the room were maintained at approximately 23~25 °C and 40~60%, respectively, with a twelve hour light/dark cycle. All of the rats were housed in stainless steel cages and were provided with deionized water to avoid the intake of extra iron.

### Sample collection and analysis

The day before they were euthanized, the SD rats were fasted overnight with free access to deionized water. Then, the rats’ body weights were recorded, and they were administered 1 mL FeSO_4_ or Fe-Gly at a relatively high dose (800 mg/L as iron). Two hours after gavage, the rats were anesthetized with chloral hydrate, and blood was collected from their eyeballs. The whole blood samples were sent to the Laboratory Animal Center of Zhejiang University for hematological measurements. Sera were separated by centrifugation at 3, 000×g for 10 min at 4 °C, and the iron levels were determined by using a serum iron assay kit (Jiancheng Bioengineering Institute, Nanjing, China).

Then, the rats were sacrificed by cervical dislocation, and liver specimens were obtained and fixed in 4% formaldehyde for immunohistochemical analysis. Approximately 3 cm of the duodenum was removed from each rat, washed with normal saline, and packed with sterile and RNase-free silver paper. After being rapidly frozen in liquid nitrogen, the samples were stored at −80 °C until RNA extraction.

### Immunohistochemical staining

The liver tissues were fixed in 4% formaldehyde and embedded in paraffin. Immunohistochemical staining to detect ferritin was performed using a DAKO Envision System (DAKO Corporation) according to the manufacturer’s protocol^49^.

Briefly, paraffin-embedded liver tissues were cut into 5 μm sections and placed on glass slides. The sections were deparaffinized with xylene, dehydrated with ethanol, and then incubated with 3% hydrogen peroxide to block endogenous peroxidase. Antigen retrieval was performed by heating the sections in 10 mM sodium citrate buffer (pH 6.0). Then, the sections were blocked with DAKO protein block (X9090; DAKO), followed by incubation with an FTL primary antibody (10727-1-AP; 1:100; Proteintech) overnight at 4 °C. Subsequently, they were incubated with the respective HRP-conjugated goat anti-rabbit (K4003; DAKO) secondary antibody for visualization of the target proteins. DAB reagent (K5007; DAKO) was applied for detection of these proteins. The tissue sections were counterstained with Aqua Hematoxylin-INNOVEX (Innovex Biosciences). Double immunohistochemistry was performed using Vina Green, according to the manufacturer’s recommendations (BioCare Medical). Liver specimens exposed to 1% bovine serum albumin instead of the respective primary antibody were used as negative controls. For the quantification of ferritin staining, 3 randomly chosen fields per section were evaluated at ×200 magnification for each sample. Image-Pro Plus 6.0 was used to determine integrated optical density (IOD) values, from which the mean density was calculated (IOD/AREA).

### RNA extraction and qualification

Total RNA was isolated from the duodenal tissues using Trizol reagent (Invitrogen), according to the manufacturer’s protocol. RNA degradation and contamination were monitored using 1% agarose gels. RNA purity was assessed using an Nanodrop ND-1000 pectrophotometer (Thermo Scientific, USA). The A260:A280 and A260:A230 ratios of each RNA sample were above 1.8 and 2.0, respectively. RNA integrity was evaluated using an Agilent 2200 TapeStation (Agilent Technologies, USA) and each sample had an RINe value of above 7.0.

### Library preparation for DGE sequencing

Three samples from each group were selected for digital gene expression measurements^50^. A total of 3 μg RNA per sample was used as the input material for the sequencing. Briefly, mRNAs were isolated from total RNA and broken into fragments of approximately 200 bp in size. Subsequently, the collected mRNAs were subjected to first-strand and second-strand cDNA synthesis, followed by adaptor ligation and low-cycle enrichment, according to the instructions of a TruSeq^®^ RNA LT/HT Sample Prep Kit (Illumina, USA). The purified products were evaluated using an Agilent 2200 TapeStation and Qubit^®^2.0 (Life Technologies, USA). They were then diluted to 10 pM for cluster generation *in situ* on a HiSeq2500 pair-end flow cell, followed by sequencing (2 × 100 bp) with a HiSeq 2500 sequencer. All sequencing data were submitted to the NCBI database under accession number SRP075016.

### Quality control and mapping analyses

Raw data (raw reads) in the FASTQ format were first processed using in-house Perl scripts. In this step, clean data were obtained by removing reads from the raw data that contained adapter sequences and ploy-N and those were low-quality. Then, the Q20, Q30 and GC content of the clean data were calculated. All downstream analyses were performed using the high-quality clean data.

The index of the reference genome was built using Bowtie v2.0.6, and single-end clean reads were aligned to the reference genome using TopHat v2.0.9^51^. TopHat was selected as the mapping tool because it generates a splice junction database based on the gene model annotation file and thus produces better mapping results than other non-splice mapping tools.

HTSeq v0.5.4p3 was used to count the number of reads mapped to each gene^52^. Then, the RPKM value of each gene was calculated based on the length of the gene and the read count mapped to it. RPKM (reads per kilobase of exon model per million mapped reads), simultaneously considers the effects of sequencing depth and gene length on the read count, and is currently the most commonly used method for the estimation of gene expression levels^53^.

The differential expression analysis of two groups (three biological replicates per group) was performed using the DESeq R package^54^. The DESeq provide statistical routines for determining differential expression genes using a model based on the negative binomial distribution^55^. The resulting P-values were adjusted using the Benjamini and Hochberg’s approach for controlling the false discovery rate^56^. Corrected P-values are also called q-values. Genes within a |log2(fold change)| >1 and q-value < 0.05 standard found by DESeq were assigned as differentially expressed.

### Functional analysis of differentially expressed genes

The GO enrichment analysis of differentially expressed genes was implemented by the GOseq R package^57^. GO terms of differentially expressed genes with q-value < 0.05 were considered significantly enriched terms.

KEGG is a database resource for understanding high-level functions and uses of the biological system (http://www.genome.jp/kegg/). The KOBAS software was used to test the statistical enrichment of differentially expressed genes in the KEGG pathways^58^.

### Quantitative real-time PCR validation

The validation was performed by using RNA from the same sample of DGE sequencing. Quantitative real-time PCR (qRT-PCR) was performed on randomly selected differentially expressed genes. Primer sequences were designed using NCBI primer designing tool (http://www.ncbi.nlm.nih.gov/tools/primer-blast/) and synthesized by Invitrogen (Thermo Fisher Scientific Inc., Shanghai, China). cDNA was synthesized with Reverse Transcriptase M-MLV (RNase H-) (TaKaRa) using the oligo dT primer. qRT-PCR with the Power SYBR^®^ Green PCR Master Mix (Applied Biosystems) was carried out on a Multiple Real-Time PCR System (Bio-Rad, America). Each sample was analyzed in triplicate and the expression of the target genes were standardized by the endogenous housekeeping gene (GAPDH). The reaction protocol comprised one cycle of 95 °C for 1 min, forty cycles of 95 °C for 15 s, 63 °C for 25 s. The gene expression was calculated by using the comparative (2^−ΔΔCt^) method^59^.

### Statistical analysis

All results are expressed as mean ± standard deviation (SD). The data were evaluated by one-way ANOVA of SPSS 22.0 (IBM). The differences between groups were assessed using Duncan’s test. P-value < 0.05 was considered statistically significant. DGE results were analyzed using R software.

## Additional Information

**How to cite this article**: Zhuo, Z. *et al*. Digital gene expression profiling analysis of duodenum transcriptomes in SD rats administered ferrous sulfate or ferrous glycine chelate by gavage. *Sci. Rep.* **6**, 37923; doi: 10.1038/srep37923 (2016).

**Publisher's note:** Springer Nature remains neutral with regard to jurisdictional claims in published maps and institutional affiliations.

## Supplementary Material

Supplementary Table S1

Supplementary Table S2

Supplementary Table S3

Supplementary Table S4

Supplementary Table S5

Supplementary Table S6

## Figures and Tables

**Table 1:** The body weights of the SD rats at 4 and 6 weeks of age.

Age in Weeks	Body Weight	
	FeSO_4_	Fe-Gly
4 w	81.5 ± 5.82	79.7 ± 7.76
6 w	191.7 ± 4.04	193.2 ± 10.34

The values are presented as the mean ± standard deviation (n = 12).

**Table 2:** Hematological parameters in the FeSO_4_ and Fe-Gly group rats.

Parameter	Unit	FeSO_4_	Fe-Gly
WBC	10^9/L	4.5 ± 0.95	4.7 ± 1.34
RBC	10^12/L	5.8 ± 0.72	5.9 ± 0.39
Hb	g/L	120.0 ± 15.72	124.7 ± 7.12
HCT	%	35.9 ± 4.93	37.1 ± 2.05
MCV	f1	61.7 ± 1.16	63.3 ± 1.75
MCH	pg	20.7 ± 0.58	21.3 ± 0.82
MCHC	g/L	335.0 ± 7.00	336.0 ± 11.78

Blood cell indices were determined for twelve SD rats in each group. White Blood Cell Count (WBC), Red Blood Cell Count (RBC), Hemoglobin Concentration (Hb), Hematocrit (HCT), Mean Corpuscular Volume (MCV), Mean Corpuscular Hemoglobin (MCH), and Mean Corpuscular Hemoglobin Concentration (MCHC). The values are presented as the mean ± standard deviation (n = 12).

**Table 3:** Serum iron-related parameters in the SD rats.

Parameter	Unit	FeSO_4_	Fe-Gly
TIBC	μmol/L	103.6 ± 14.23	99.1 ± 14.66
SI	μmol/L	45.7 ± 5.12	66.7 ± 12.72*
TAST	%	45 ± 11.3	67 ± 9.0*

Total Iron Banding Capacity (TIBC), Serum Iron (SI), and Transferrin Saturation (TAST). The values are presented as the mean ± standard deviation (n = 12).

^*^Indicates a significant difference in the mean value between the two groups at P < 0.05.

**Table 4:** Summary of sequencing analysis.

Item	C1	C2	C3	T1	T2	T3
Raw reads	20,446,968 (100%)	20,983,958 (100%)	23,325,694 (100%)	21,951,812 (100%)	21,586,433 (100%)	22,458,258 (100%)
adaptor sequences	385,382 (1.88%)	167,806 (0.80%)	22,261 (0.10%)	16,393 (0.07%)	19,553 (0.09%)	6,456 (0.03%)
ambiguous sequences	797 (<0.01%)	873 (<0.01%)	1,000 (<0.01%)	939 (<0.01%)	880 (<0.01%)	997 (<0.01%)
low-quality sequences	340,686 (1.67%)	400,509 (1.91%)	404,448 (1.73%)	384,338 (1.75%)	393,886 (1.82%)	394,657 (1.76%)
Clean reads	19,720,103 (96.45%)	20,414,770 (97.29%)	22,897,985 (98.17%)	21,550,142 (98.17%)	21,172,114 (98.08%)	22,056,148 (98.21%)
clean_Q20	98.95%	98.93%	98.96%	98.95%	98.92%	98.95%
clean_Q30	95.53%	95.42%	95.55%	95.53%	95.38%	95.52%

C1, C2, and C3: control group, namely the FeSO4 group; T1, T2, and T3: treatment group, namely the Fe-Gly group. Q20: the percentage of bases with a Phred value > 20; and Q30: the percentage of bases with a Phred value > 30.

**Table 5:** The data for the sequencing reads that mapped to the reference genome.

Mapping Statistics	C1	C2	C3	T1	T2	T3
Effective reads	19,720,103 (100%)	20,414,770 (100%)	22,897,985 (100%)	21,550,142 (100%)	21,172,114 (100%)	22,056,148 (100%)
Total mapped	18,873,056 (95.70%)	19,518,820 (95.61%)	21,935,403 (95.80%)	20,668,869 (95.91%)	20,209,758 (95.45%)	21,191,619 (96.08%)
Multiple mapped	937,908 (4.76%)	1,236,043 (6.05%)	1,093,573 (4.78%)	1,041,601 (4.83%)	1,122,271 (5.30%)	1,194,494 (5.42%)
Uniquely mapped	17,935,148 (90.95%)	18,282,777 (89.56%)	20,841,830 (91.02%)	19,627,268 (91.08%)	19,087,487 (90.15%)	19,997,125 (90.66%)
Reads mapped to ‘+’	9,326,071 (47.29%)	9,611,103 (47.08%)	10,844,189 (47.36%)	10,227,148 (47.46%)	9,958,528 (47.04%)	10,452,084 (47.39%)
Reads mapped to ‘−’	9,546,985 (48.41%)	9,907,717 (48.53%)	11,091,214 (48.44%)	10,441,721 (48.45%)	10,251,230 (48.42%)	10,739,535 (48.69%)

C1, C2, and C3: control group, namely the FeSO4 group; T1, T2, and T3: treatment group, namely the Fe-Gly group. “ + ” refers to the sense strand, and “−” refers to the anti-sense strand.

**Table 6:** The main genes related to iron metabolism and their expression differences between the two groups.

Metabolic process	Gene	Function	Illumina mRNA-seq (log2 fold change)
cellular iron/heme uptake	Tf	Plasma, lymph, and CSF ferric iron carrier	0.09
	Tfrc	Facilitates Tf-dependent iron uptake	−0.79
	TfR2	Regulates hepcidin; mediates Tf-bound and non-transferrin-bound iron uptake	0.21
	Steap3	Erythroid endosomal ferrireductase	−0.08
	Slc11a2	Endosomal ferrous iron transporter; transmembrane non-transferrin iron transporter	0.15
	Cybrd1	Intestinal ferrireductase	0.21
	Slc39a4	Non-transferrin iron transporter in liver	0.38
	Tim2	H-ferritin receptor	0.28
	Scara5	L-ferritin receptor	0.18
	FLVCR2	Heme importer	0.70
	Slc46a1	Intestinal heme transporter	0.37
	Hmox1	Heme iron reutilization	1.55*
	Hmox2	Heme iron reutilization	0.10
	Lcn2	Facilitator of non-transferrin iron import	0.21
cellular iron storage	Fth1	Iron storage protein subunit; ferroxidase	0.11
	Ftl	Iron storage protein subunit	0.05
intracellular iron trafficking	Fech	Insertion of iron into porphyrin	−0.12
	Alas2	Heme biogenesis	1.22
	Abcb10	Interacts with Fech and Mitoferrin-1; transport ligand unknown	−0.12
	Abcb7	Interacts with Fech; cytosolic ISC maturation	−0.01
	Abcb6	Import of porphyrin into mitochondria	0.24
	Slc25a37	Mitochondrial iron import	−0.08
	Bdh2	Siderophore biosynthesis	0.62
cellular iron/heme export	Abcg2	Partially exports heme; PPIX exporter	0.36
	Slc40a1	Regulates efflux of iron from cells	0.01
cellular iron balance	Aco1	Post-transcriptional regulation of target mRNAs via IREs; cytosolic aconitase	0.09
	Ireb2	Post-transcriptional regulation of target mRNAs via IREs	−0.12

^*^Indicates a significant difference in gene expression at q-value < 0.05. Fold change = Fe-Gly group (mean)/FeSO_4_ group (mean); the “mean” is the mean of three biological replicates.
