# Supplementary material for: Digital gene expression profiling analysis of duodenum transcriptomes in SD rats administered ferrous sulfate or ferrous glycine chelate by gavage
Source: Sci Rep. 2016 Nov 30;6:37923. doi: 10.1038/srep37923 (PMC5128800; doi:10.1038/srep37923)
Supplement: Supplementary Table S5 [file srep37923-s5.doc]

**Digital gene expression profiling analysis of duodenum transcriptomes in SD rats administered ferrous sulfate or ferrous** **glycine chelate by gavage**

**Zhao Zhuo, Shenglin Fang, Qiaoling Hu, Danping Huang, Jie Feng^*^**

Key Laboratory of Molecular Animal Nutrition, Ministry of Education, College of Animal Science, Zhejiang University, Hangzhou, 310058, P.R. China

*For correspondence: fengj@zju.edu.cn

**Table S5 The primers of qRT-PCR**

| Gene | Genbank Accession | Primer Sequences (5'to3') | Size (bp) | Annealing (℃) |
| --- | --- | --- | --- | --- |
| Cyp2b1 | NM_001134844.1 | GTCAGGGGACACCCAAAGTC  GGTGTCTGTCCCACATAGCA | 192 | 60 |
| Mt1a | NM_138826.4 | TGTCGCTTACACCGTTGCT  AGGTGCATTTGCAGTTCTTGC | 122 | 60 |
| Slc34a2 | NM_053380.2 | CCTGTGGCTGGACTTGTGAT  TCTCCTGCCTGCATAAGTGC | 191 | 60 |
| Pck1 | NM_198780.3 | GACAGACTCGCCCTATGTGG  AGGCCCAGTTGTTGACCAAA | 155 | 60 |
| Duox2 | NM_024141.1 | CCAGCAAGTACGAGAGGCTCTGA  CCTTGTCGGCCAGAGAAAACA | 136 | 63 |
| Msmo1 | NM_080886.1 | CATTGGAATTGTGCTTTTGTGTGA  CAACGGGTTGAGAGGGATATCAT | 115 | 63 |
| G6pc | NM_013098.2 | CTCCAGGCCGAGGGTAAAAG  TGGCTACTCATTACACGGGC | 196 | 60 |
| Hmox1 | NM_012580.2 | TGCACATCCGTGCAGAGAAT  CTGGGTTCTGCTTGTTTCGC | 147 | 60 |
| Reg3b | NM_053289.1 | AAGATGTTGCATCGCTTGGC  CCTTTGGGGCAACTAATGCG | 131 | 60 |
| GAPDH | NM_017008.4 | TGTGGATCTGACATGCCGC  TCCAGGGTTTCTTACTCCTTGG | 294 | 60 |
