# Supplementary material for: Digital gene expression profiling analysis of duodenum transcriptomes in SD rats administered ferrous sulfate or ferrous glycine chelate by gavage
Source: Sci Rep. 2016 Nov 30;6:37923. doi: 10.1038/srep37923 (PMC5128800; doi:10.1038/srep37923)
Supplement: Supplementary Table S6 [file srep37923-s6.doc]

**Digital gene expression profiling analysis of duodenum transcriptomes in SD rats administered ferrous sulfate or ferrous** **glycine chelate by gavage**

**Zhao Zhuo, Shenglin Fang, Qiaoling Hu, Danping Huang, Jie Feng^*^**

Key Laboratory of Molecular Animal Nutrition, Ministry of Education, College of Animal Science, Zhejiang University, Hangzhou, 310058, P.R. China

*For correspondence: fengj@zju.edu.cn

**Table S6** Composition and nutrient levels of basal diets

| Ingredients | Percentage | Ingredients | Percentage |
| --- | --- | --- | --- |
| Corn Starch | 39.75 | Mineral Mix | 3.50 |
| Casein Lactic | 20.00 | Vitamin Mix | 1.00 |
| Granular Sugar | 10.00 | L-Cystine | 0.30 |
| Dextrin | 13.20 | Choline Bitartrate | 0.25 |
| Solka Floc-40 | 5.00 | Soy Oil | 7.00 |

Supplied mineral mix contained (based on total diet): Calcium 0.52 %, Phosphorus 0.20%, Potassium 0.38%, Sodium 0.11%, Magnesium 0.05%, Iron 52.02 ppm, Zinc 36.76 ppm, Manganese 11.33 ppm, Copper 6.73 ppm, Cobalt 0.02 ppm, Iodine 0.21 ppm.

Supplied vitamin mix contained (based on total diet): Vitamin A 4.00 IU/g, Vitamin D_3_ 1.00 IU/g, Alpha-Tocopherol 75.00 IU/kg, Thiamine 5.00 ppm, Ribofl avin 6.0 0 ppm, Niacin 30.00 ppm, Pantothenic Acid 15.00 ppm, Choline 1000.00 ppm, Pyridoxine 6.00 ppm, Folic Acid 2.00 ppm, Biotin 0.20 ppm, Vitamin B_12_ 25.00 mcg/kg, Vitamin K 0.90 ppm.
